# Importance of the Preservation of the Teeth

**Published:** 1851-01

**Authors:** L. S. Parmly


### ARTICLE VIII.

*Importance of the Preservation of the Teeth.* By L. S. PARMLY, M. D.

THE teeth and gums, it is well known, are organs most important to the animal economy. Their agency in distinct articulation, in ministering to the comfort and promoting the health of the individual, is too obvious to be insisted on. Suffice it to observe, that when, from disease or accident, impediments arise to interrupt the discharge of their proper functions, calamities most mischievous to the system ensue. Every medical man knows that imperfect mastication is a fruitful cause of many disorders, the most distressing, dangerous and even fatal to the human frame, and that for the proper performance of this operation of the economy, healthy teeth are necessary. The fact is also equally well established, that the means recommended for the preservation of the health of these organs and their immediate connections, are insufficient for the accomplishment of this object. But that it may be done, an experience of forty years has furnished the writer with the most abundant and ample proof. As it regards health and cleanliness, the means used, are for self-preservation. For the teeth and gums, waxed floss silk, a dental polisher and tongue scraper, used daily, will prevent disease of these organs, offensive breath and dilapidated mouths.

In examining the progress of improvements in the varied branches of surgery, we may observe, with regret, that until recently, the dental department has not kept pace with many others of less utility. It still remains to point out to society the importance of preventing diseases of the teeth, gums and their connexions, in almost every instance, with the general health and preservation of the human system. Nothing can tend more to accomplish this object, than to embody a thorough hygienic course of instruction in the education of those who are preparing for this branch of practice.

The wants of general society, as well as the disposition of the present period, so favorable to the universal diffusion of knowledge, loudly calls for information upon this subject. When we advert to the progress of medical and surgical science, we are struck with the rapid and beneficial changes which have taken place in conducting those enlightened principles, which are the fruits of liberal education and the peculiar spirit of the present age.

The consequence of this will be, that the prejudices of the nurse, the mysteries of the empyric, and the pedantry of the schools will be lost sight of, as the years beyond the flood, and then the civilized nations will swarm with dental surgeons and hygienists, from which governments will select well qualified dentists for the navy, army and all public institutions of charity and learning.

Why the public are not sufficiently acquainted with the numberless and painful afflictions which originate in a neglected mouth, is self-evident. But, when the people are fully convinced, that from this cause alone, numbers drag out a life of distress, they will feel a greater curiosity, and take a more lively interest, in obtaining information respecting the dental art, which the considerations of appearance, comfort and health render so essential.

That such an interest has not been felt, is the more astonishing, since we cannot but observe how anxiously solicitous the mind daily discovers itself to be, on subjects of less importance and utility.

The truth of this observation is obvious, for, in most instances, it is either the loss of the teeth, or the sensation of pain in the mouth, that calls the attention to its condition, when they readily discover the cause which moulders them away like other species of rusting, affecting the contiguous parts, tainting the breath, and rendering it disagreeable even to the individual himself. Thus, daily experience proves that the teeth and gums are generally the seat of pain and disease, and the question to be considered is, whether this state of infirmity be naturally entailed upon them, or is the result of ignorance and neglect.

The dental organism constitutes the natural ornament and armory of the mouth, and may be regarded as a weapon of defence. The teeth of the uncivilized nations are generally regular and free from disease, and no accumulations form to deface them. But, in civilized societies, with so much crossing, and the refinements in the culinary art, give the food a greater tendency to acquire noxious power, and form chemical combinations. This, with the modern help of knives and forks, lessens the friction which they would otherwise receive in the act of mastication. The more we examine nature in the policy of the animal machine, however varied and multiplied its parts, the more we discover the intimate connection of each part with the whole.

Numerous as they are, to each is assigned some peculiar and needful office, and in the healthy state the most perfect harmony subsists between them; no one obstructs, but each assists the operation of the other, and thus promotes the ultimate preservation, and gives longevity to the machine. By this wise adjustment, there is no schism in the body, no separate or interfering ends are pursued by the multiplicity of its members, but the safety and support of each are the undivided care of all. In this view, there is no part of the frame-work of man that is not of importance, however trifling or insignificant it may appear. The most vital, as well as those on which the lesser energies of the system depend, are equally essential to life and its comforts. On these considerations, the dental art claims an important rank in the care of the human structure, for on the teeth depends the proper and natural expression of the face; by their loss, the character and symmetry of the features are destroyed, and beauty is deprived of its chief attraction and principal charm.

To the teeth also is assigned the chief power of enunciation. If the great and pre-eminent prerogative of man is the possession of speech, it can never be complete or perfect without the teeth to modulate the sound. This circumstance gives them additional value to those who wish to shine either in the senate, the pulpit, at the bar, or upon the stage. Without these instruments of utterance, the graces of their eloquence is partially lost, and the power of affecting the mind and convincing the understanding is considerably diminished.

It is the loss of the teeth that produces the leading marks of age, and occasions the contracted countenance, the wrinkles of the face, and those unseemly changes which youth and beauty ever wish to see at a distance.

In order to secure perfect health, it is necessary that the food be sufficiently comminuted by the action of the teeth. By this operation it becomes blended with the saliva, thus rendering its solution easy when it descends into the stomach, to be mixed with the gastric juice, without any other additional fluid to render its digestion complete, in forming it into perfect chyme.

The enamel covering the crowns of the teeth may be considered as an extraneous body, being void of perspiration or circulation; hence it does not possess the power of freeing itself from injurious accumulations; therefore dental hygiene becomes an indispensable part of education, for promoting health and comfort, and a primary duty, in view of the entire prevention of disease and deformity of the face.

If to vaccination we owe, of late years, a decrease of the annual partial deformity and mortality, a point clearly proved, the prevention of dental disease will add to this decrease, for the constitution will not be interrupted unseasonably, or the health impaired by sleepless nights and days of tormenting pain and agitation, as happens to the majority of civilized men, who date the first symptoms of disease to their negligence in not regarding the health of the mouth and teeth, which may be done under every variety of constitution, climate and habit of body. Should these suggestions have the effect of awakening the profession to the importance of the prevention of disease in the dental organism, and of inducing them to impart the necessary instruction for the accomplishment of this object, to those who may seek their advice, I shall not regret having furnished this article, founded on my experience.

Having spent a few months in Baltimore, during the present session of the Dental College, I am gratified to find that the hygiene of the mouth constitutes a portion of the instruction given in this valuable Institution. I am also happy to learn that an effort is about to be made for carrying out this feature of instruction on a still more extensive plan, in aiding that virtue.

### ARTICLE IX.

*Operations on the Antrum.* By R. D. ADDINGTON, D. D. S.

